# Electronic health records accurately predict renal replacement therapy in acute kidney injury

**DOI:** 10.1186/s12882-019-1206-4

**Published:** 2019-01-31

**Authors:** Sanmay Low, Anantharaman Vathsala, Tanusya Murali Murali, Long Pang, Graeme MacLaren, Wan-Ying Ng, Sabrina Haroon, Amartya Mukhopadhyay, Shir-Lynn Lim, Bee-Hong Tan, Titus Lau, Horng-Ruey Chua

**Affiliations:** 1 Division of Nephrology, University Medicine Cluster, National University Hospital, Level 10 Medicine Office, NUHS Tower Block, 1E Kent Ridge Road, Singapore, 119228 Singapore; 2 Renal Unit, Department of Medicine, Ng Teng Fong General Hospital, Singapore, Singapore; 3 Department of Medicine, Yong Loo Lin School of Medicine, National University of Singapore, Singapore, Singapore; 4 Biostatistics, Saw Swee Hock School of Public Health, National University of Singapore, Singapore, Singapore; 5 National University Heart Centre, National University Hospital, Singapore, Singapore; 6 Division of Respiratory and Critical Care Medicine, University Medicine Cluster, National University Hospital, Singapore, Singapore; 7 Department of Anaesthesia, National University Hospital, Singapore, Singapore

**Keywords:** Acute kidney injury, Decision support techniques, Electronic health records, Epidemiology, Mortality, Outcomes and process assessment, Renal replacement therapy

## Abstract

**Background:**

Electronic health records (EHR) detect the onset of acute kidney injury (AKI) in hospitalized patients, and may identify those at highest risk of mortality and renal replacement therapy (RRT), for earlier targeted intervention.

**Methods:**

Prospective observational study to derive prediction models for hospital mortality and RRT, in inpatients aged ≥18 years with AKI detected by EHR over 1 year in a tertiary institution, fulfilling modified KDIGO criterion based on serial serum creatinine (sCr) measures.

**Results:**

We studied 3333 patients with AKI, of 77,873 unique patient admissions, giving an AKI incidence of 4%. KDIGO AKI stages at detection were 1(74%), 2(15%), 3(10%); corresponding peak AKI staging in hospital were 61, 20, 19%. 392 patients (12%) died, and 174 (5%) received RRT. Multivariate logistic regression identified AKI onset in ICU, haematological malignancy, higher delta sCr (sCr rise from AKI detection till peak), higher serum potassium and baseline eGFR, as independent predictors of both mortality and RRT. Additionally, older age, higher serum urea, pneumonia and intraabdominal infections, acute cardiac diseases, solid organ malignancy, cerebrovascular disease, current need for RRT and admission under a medical specialty predicted mortality. The AUROC for RRT prediction was 0.94, averaging 0.93 after 10-fold cross-validation. Corresponding AUROC for mortality prediction was 0.9 and 0.9 after validation. Decision tree analysis for RRT prediction achieved a balanced accuracy of 70.4%, and identified delta-sCr ≥ 148 μmol/L as the key factor that predicted RRT.

**Conclusion:**

Case fatality was high with significant renal deterioration following hospital-wide AKI. EHR clinical model was highly accurate for both RRT prediction and for mortality; allowing excellent risk-stratification with potential for real-time deployment.

**Electronic supplementary material:**

The online version of this article (10.1186/s12882-019-1206-4) contains supplementary material, which is available to authorized users.

## Background

Life expectancy has steadily increased worldwide, in part due to the improvement in healthcare standards. [1] National census in Singapore showed that 12% of the population was aged 65 years and above in 2016 [2]. In this elderly cohort, the hospital admission rate was more than 200 per 1000 resident-population, [3] and they suffer from complex illnesses. Acute kidney injury (AKI) can manifest as part of their global clinical deterioration from a multitude of acute diseases, for which they might have otherwise not survived previously [4]. Accordingly, the incidence of AKI has risen in parallel with increasing case complexity and ageing population despite medical advances, and is a global public health concern [5, 6].

In that regard, patients with AKI in need of renal replacement therapy (RRT) have prolonged hospitalization and high mortality in excess of 40% [7, 8]. These data, however, are described in the setting of critical care, which only represents a snapshot of the AKI burden hospital-wide, the vast majority of which may evolve outside of the critical care environment and involve different clinical trajectories. A national chronic disease registry captures the census of end-stage kidney disease (ESKD) with an initial 90-day censorship, [9] but the impact of AKI-RRT remains unclear. The advent of EHR can now provide a wealth of clinical data to be routinely studied, with linked biochemistry and serial serum creatinine (sCr) measurements that facilitate the translation of consensus AKI definitions to electronic AKI detection [10, 11]. Multiple data-points may be used to formulate an electronic risk assessment tool, to predict AKI patients at highest risk of RRT or mortality, thereby allowing clinicians a potential 24–48 h window to optimize management pre-emptively.

In this study, our objective was to analyze EHR data to formulate risk prediction models for RRT and mortality in hospital-wide patients with AKI. We aimed to examine our local in-hospital AKI epidemiology, not confined to critical care, using EHR to demonstrate the feasibility of such an analytic approach.

We studied incidences and predictors of RRT and mortality in these patients. We hypothesized that the wealth of clinical data made possible by EHR, ranging from demographics, comorbidities, biochemistry, and acute illnesses, can be incorporated into accurate prediction tools for adverse AKI outcomes.

## Methods

### Study design and setting

We performed a prospective observational study in a tertiary institution with 1200 acute beds, involving patients aged 18 years or more, hospitalized from November 2015 till October 2016, with AKI detected by methods using EHR.

### Study population

Patients who fulfilled electronic AKI definition had their details entered into the database. Only the first AKI episode during the study period for every unique patient was included. The exclusion criteria included: (i) patients with advanced chronic kidney disease (CKD) as defined by baseline estimated glomerular filtration rate (eGFR) of less than 15 mL/min/1.73 m^2^, using the CKD Epidemiology Collaboration (CKD-EPI) equation; [12] (ii) patients with RRT in previous hospitalizations and failed to recover kidney function to a current eGFR of more than 30 mL/min/1.73 m^2^; and (iii) patients with ESKD.

### EHR AKI definitions and staging

We followed the KDIGO AKI criteria using serial sCr measures, [13] but with modification made for the timing of baseline sCr. We accepted a baseline sCr within one year prior to the AKI-defining sCr, instead of the proposed 7-day window for the relative sCr change criterion. The latter may not be a practical criterion for patients with community-acquired AKI, who would not have had such frequent blood-test surveillance performed as outpatient. Patients’ sCr measures performed in National University Health System Singapore’s or National Healthcare Group Singapore’s primary to tertiary care facilities, were made available for analysis. Creatinine was measured using Advia 2400 (Siemens, Munich, Germany) enzymatic creatinine method traceable to isotope-dilution mass spectrometry standard. Patients who met either the relative change criteria of at least 1.5 times increase in sCr over baseline, or absolute increase in sCr by at least 26.5 μmol/L (0.3 mg/dL) within a 48-h window, were detected by EHR as having AKI. The timing of this AKI-defining sCr, and onset in days from hospital admission were recorded. Community-acquired AKI was defined by AKI onset within 48 h from hospital admission, while hospital-associated AKI was implied by onset after 48 h, as inferred from recent literature on AKI epidemiology [14].

**Fig. 1:**
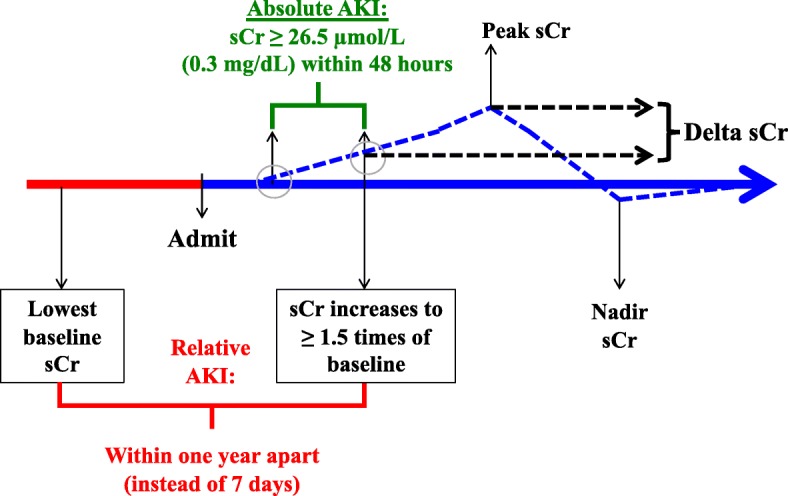
Chart diagram depicting modified KDIGO criterion used for AKI detection. Relative AKI detected (red) when sCr increases to ≥1.5x that of patient’s lowest baseline sCr in the past one year. Absolute AKI detected (green) with an increase in sCr of ≥26.5 μmol/L (0.3 mg/dL) within 48 h. Delta sCr is the rise from in sCr from AKI detection till peak sCr. sCr = serum Creatinine

Patients who entered the database had their peak sCr and nadir sCr during the same hospitalization tracked. The nadir sCr served as a surrogate for baseline sCr, for patients who entered the database using the absolute sCr change criteria. We computed the delta sCr, which was the difference between the peak sCr and the AKI-defining sCr, as a measure of AKI trajectory. A chart diagram of this modified AKI criteria is shown in Fig. 1.

We defined the AKI stages 1, 2 or 3 according to proposed KDIGO criteria [13], at the point of AKI detection by EHR, and subsequent peak AKI severity during hospitalization, based on serial sCr change and need for RRT.

### Electronic clinical data sources

The study subjects’ relevant clinical data that were pre-selected by the study investigators and data engineers were consolidated into a database. The 32 EHR variables included were classified into 4 categories, namely: (i) patient demographics, (ii) comorbidities, (iii) kidney function indices, and (iv) acute disease categories (Fig. 3 legend). Our EHR sources included: (i) Computerized Patient Support System 2 (CPSS2), which was developed locally by Integrated Health Information System Pte Ltd., and (ii) System Application and Product (SAP GUI for Windows, copyright SAP 1993–2010). Patients’ acute disease diagnosis codes were based on ICD-9-CM (International Classification of Diseases, Ninth Revision, Clinical Modification) up till 2015, and SNOMED-CT (Systematized Nomenclature of Medicine - Clinical Terms) after 2015. We classified these diagnoses into 10 categories (see RESULTS and Additional file 1: Table S1). These acute diseases were not mutually exclusive, and each patient may have one or more acute illnesses during the index hospitalization.

### Study outcomes

Primary outcome studied was hospital mortality. Secondary outcomes included: (i) RRT, which was obtained from billing codes for various modalities ranging from intermittent hemodialysis and continuous RRT, (ii) deterioration to subsequent ICU transfer, and (iii) duration of hospitalization from time of AKI detection.

### Statistical analysis

We described the incidences of hospital mortality and RRT among these patients with AKI and made univariate comparisons of the electronic clinical variables between the deceased versus survivors, and RRT versus non-RRT patients. Parametric variables and non-parametric variables were presented in mean (±standard deviation), and median (interquartile range) and compared using t-test and Wilcoxon rank-sum test, respectively. Categorical variables were presented in frequency (percentage) and compared using Chi-square or Fisher-exact test where appropriate.

Multivariate logistic regression was used to assess factors associated with mortality and RRT from the list of 32 EHR variables with temporal restrictions on variable inclusion (Fig. 3). We included all 32 variables for the mortality prediction model, but only 23 variables for the RRT prediction model, excluding all variables under “acute diagnosis categories”, as these acute diagnoses may have happened before or after initiation of RRT. A two-sided *p* < 0.05 was taken as measure of statistical significance. The model performance for mortality and RRT prediction was assessed by the derived area under receiver operating characteristics (AUROC) curve. Finally, a decision tree analysis was performed. The trees were built using a binary recursive partitioning algorithm with rpart package in R. Decision tree model accuracy was derived for performance assessment if a binary tree could be constructed. Ten-fold cross-validation was applied to logistic regression models for out of sample AUROC, and the balanced accuracy was reported for decision tree model. We also performed multivariate analysis using the same 32 EHR variables, to compare factors associated with mortality between patients with mild AKI (peak KDIGO stage 1) and patients with more severe AKI (peak KDIGO stages 2 and 3). All analyses were performed with R Statistical Software version 3.3.1 (Vienna, Austria).

## Results

### Patient profile

**Fig. 2:**
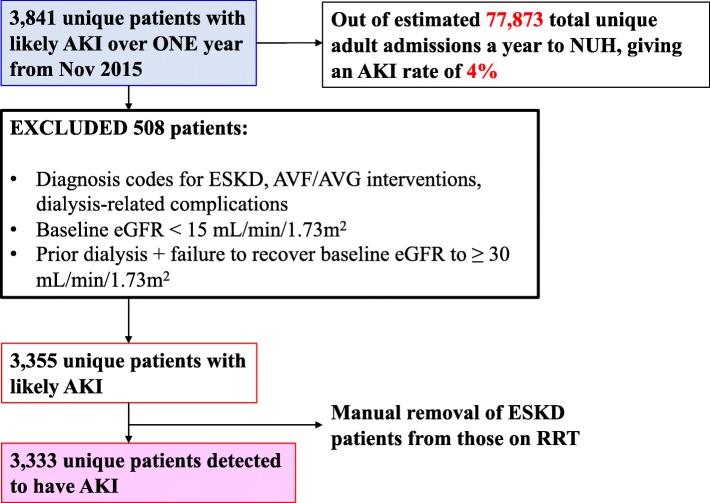
Flow diagram depicting patient recruitment numbers and exclusion criteria. ESKD = End Stage Kidney Disease, AVF = Arteriovenous fistula, AVG = Arteriovenous graft, RRT = Renal Replacement Therapy

We collected data from 3841 unique patients whose sCr trajectories triggered the diagnosis of AKI. Following application of the exclusion criteria, there were 3333 patients diagnosed with AKI (Fig. 2). This occurred in an estimated 77,873 unique patient admissions over the study period involving patients of similar age profile, giving an AKI rate of 4%. The patient profile is shown in Table 1.

**Table 1:** Patient profile and subgroup comparisons (mortality versus survivors); AND (RRT versus no RRT)

Variables	Entire Cohort		Hospital Mortality					RRT				
			Survivors		Mortality		*p*-value	No RRT		Received RRT		*p*-value
	*n* = 3333		*n* = 2941		*n* = 392			*n* = 3159		*n* = 174		
Demographics												
Age, mean (SD), yrs	65	(16)	65	(16)	70	(15)	< 0.0001	65	(16)	64	(15)	0.25
Male gender, No. (%)	1815	(54)	1607	(55)	208	(53)	0.56	1701	(54)	114	(66)	0.003
Comorbidities, No. (%)												
Diabetes mellitus	1206	(36)	1088	(37)	118	(30)	0.008	1131	(36)	75	(43)	0.05
Hypertension	1532	(46)	1370	(47)	162	(41)	0.05	1445	(46)	87	(50)	0.27
Ischemic heart disease	486	(15)	418	(14)	68	(17)	0.10	430	(14)	56	(32)	< 0.001
Heart failure	547	(16)	484	(16)	63	(16)	0.85	513	(16)	34	(20)	0.25
Peripheral vascular disease	123	(4)	114	(4)	9	(2)	0.12	114	(4)	9	(5)	0.29
Cerebrovascular disease	415	(12)	350	(12)	65	(17)	0.008	398	(13)	17	(10)	0.27
Liver cirrhosis	153	(5)	125	(4)	28	(7)	0.01	143	(5)	10	(6)	0.45
Solid organ malignancy	509	(15)	402	(14)	107	(27)	< 0.001	497	(16)	12	(7)	0.002
Haematological malignancy	167	(5)	149	(5)	18	(5)	0.69	154	(5)	13	(7)	0.13
Baseline eGFR < 60 mL/min/1.73 m^2^	787	(24)	700	(24)	87	(22)	0.48	727	(23)	60	(34)	0.001
AKI-related variables												
Relative criterion (vs absolute), No. (%)	3213	(96)	2835	(96)	378	(96)	0.97	3041	(96)	172	(99)	0.09
AKI onset days from admission, median (IQR)	1.3	(0.5–4.6)	1.2	(0.5–4.2)	1.5	(0.6–6.4)	0.003	1.3	(0.5–4.6)	1.1	(0.4–3.5)	0.29
Hospital-associated AKI (vs community-acquired), No. (%)	1293	(39)	1119	(38)	174	(44)	0.02	1233	(39)	60	(34)	0.23
Serum biochemistry at AKI detection, median (IQR)												
Sodium, mmol/L	136	(133–139)	136	(134–139)	136	(132–141)	0.28	136	(133–139)	136	(133–140)	0.82
Potassium, mmol/L	4.1	(3.7–4.5)	4.1	(3.7–4.5)	4.2	(3.8–4.7)	0.0003	4.1	(3.7–4.5)	4.4	(4.0–5.0)	< 0.0001
Urea, mmol/L	9.2	(6.1–14.3)	8.9	(5.9–13.6)	13.0	(8.4–19.2)	< 0.0001	9.1	(6.0–14.0)	13.1	(8.7–18.4)	< 0.0001
Creatinine, μmol/L	122	(85–184)	121	(84–182)	131	(95–206)	0.001	119	(84–178)	201	(144–319)	< 0.0001
Delta serum creatinine, median (IQR), μmol/L	0	(0–11)	0	(0–7)	10	(0–77)	< 0.0001	0	(0–8)	116	(21–225)	< 0.0001
RRT, No. (%)	174	(5)	98	(3)	76	(19)	< 0.001					
Admission details, No. (%)												
Medical (vs surgical) specialties	2314	(69)	1991	(68)	323	(82)	< 0.001	2201	(70)	113	(65)	0.19
AKI detected in ICU	418	(13)	298	(10)	120	(31)	< 0.001	333	(11)	85	(49)	< 0.001
Acute disease categories, No (%)												
Pneumonia	329	(10)	241	(8)	88	(22)	< 0.001	296	(9)	33	(19)	< 0.001
Urinary tract infection	185	(6)	166	(6)	19	(5)	0.52	182	(6)	3	(2)	0.02
Intraabdominal infection	124	(4)	110	(4)	14	(4)	0.87	117	(4)	7	(4)	0.83
Musculoskeletal infection	159	(5)	154	(5)	5	(1)	0.001	153	(5)	6	(3)	0.40
Acute cardiac diseases	596	(18)	509	(17)	87	(22)	0.02	544	(17)	52	(30)	< 0.001
Hepatic decompensation	98	(3)	73	(2)	25	(6)	< 0.001	89	(3)	9	(5)	0.07
Acute ischemic stroke	109	(3)	87	(3)	22	(6)	0.006	104	(3)	5	(3)	0.76
Intracranial haemorrhage (non-traumatic)	99	(3)	93	(3)	6	(2)	0.07	99	(3)	0	(0)	0.02
Other secondary outcomes												
Total patients who received ICU care, No. (%)	1290	(39)	1066	(36)	224	(57)	< 0.001	1134	(36)	156	(90)	< 0.001
Hospitalization days from AKI onset, median (IQR)	7.6	(4.0–15.8)	7.6	(4.1–15.9)	7.8	(3.0–15.3)	0.07	7.3	(3.9–15.0)	16.3	(8.7–35.3)	< 0.0001

*AKI* Acute kidney injury; *eGFR* Estimated glomerular filtration rate by CKD-EPI Equation; *ICU* Intensive care unit; *IQR* Interquartile range; *RRT* Renal replacement therapy; *SD* Standard deviation

### Outcomes

Hospital mortality occurred in 392 of 3333 patients (12%), and 174 of 3333 patients (5%) received RRT. KDIGO staging on diagnosis of AKI were 1(74%), 2(15%), and 3(10%); corresponding peak AKI staging in hospital were 61, 20, and 19%. 418 patients (13%) had their AKI onset in ICU, and a further 872 patients deteriorated and received ICU care (see Additional file 2: Figure S1).

Patients who died (versus survived) were observed to be older (70 versus 65 years old, *p* < 0.0001), with more comorbidities such as solid organ malignancy (27% versus 14%, *p* < 0.001), cerebrovascular disease (17% versus 12%, *p* = 0.008), and liver cirrhosis (7% versus 4%, *p* = 0.01); more had hospital-associated AKI (44% versus 38%, *p* = 0.02) and were from medical (versus surgical) specialties (82% versus 62%, p < 0.001), and more had AKI onset in ICU (31% versus 10%, p < 0.001). More patients who died also suffered from pneumonia (22% versus 8%, p < 0.001), acute cardiac diseases (22% versus 17%, p = 0.02), hepatic decompensation (6% versus 2%, p < 0.001), and acute ischemic stroke (6% versus 3%, *p* = 0.006) (see Table 1).

More patients who received RRT (versus none) were males, and more had ischemic heart disease (IHD), baseline eGFR < 60 mL/min/1.73m^2^, and AKI onset in ICU. More RRT patients also suffered from pneumonia and acute cardiac diseases. On the other hand, fewer RRT (versus no RRT) patients had solid organ malignancy (all *p* < 0.05). Patients who received RRT had more than double the median hospitalization duration from AKI onset, versus those with no RRT (*p* < 0.0001, see Table 1).

### Multivariate analyses for mortality and RRT

**Fig. 3 a:**
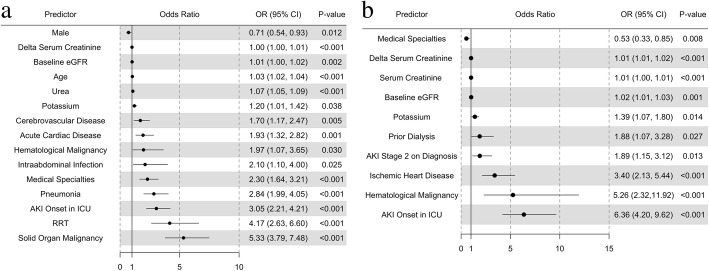
(Predictors of Hospital Mortality), **Fig**. **3b** (Predictors of RRT) Forest plot of multivariate logistic regression showing independent predictors of hospital mortality (Fig. 3a) and RRT (Fig. 3b). 32 EHR variables studied for mortality, and 23 variables for RRT: Demographics [4]: Age, Gender, Medical or Surgical specialties, ICU status on initial AKI diagnosis; Co-morbidities [9]: DM, Hypertension, IHD, PVD, CCF, Liver cirrhosis, Cerebrovascular disease, Solid organ malignancy, Haematological malignancy; Kidney function indices [11]: Baseline eGFR, AKI onset days from admission, Hospital-associated or community-acquired AKI, Biochemistry on AKI diagnosis including serum sodium, potassium, urea, creatinine levels, KDIGO stage 2 or 3 (vs 1) on AKI diagnosis, Delta-serum creatinine, Prior dialysis, Current need for RRT; Acute disease categories – Excluded in RRT prediction model [8]: Pneumonia, Intraabdominal infection, MSK infection, UTI, Acute Cardiac Diseases, Hepatic decompensation, Acute ischaemic stroke, Non-traumatic intra-cranial haemorrhage. DM = Diabetes Mellitus, IHD = Ischaemic Heart Disease, PVD = Peripheral vascular disease, CCF = Congestive Cardiac Failure

The results of the multivariate logistic regression models and distribution of odds ratio are shown in Fig. 3. 15 of 32 clinical variables studied were independently associated with hospital mortality (Fig. 3a). Subgroup analysis was performed to identify which of these 15 variables remained significant for mortality prediction in patients with more severe AKI (KDIGO peak stage 2 or 3), and not in patients with mild AKI. They included admission under a medical specialty, higher baseline eGFR, higher serum potassium level on AKI diagnosis, higher delta-sCr, presence of intraabdominal infections, acute cardiac diseases, and hepatic decompensation (all *p* < 0.05, see Additional file 3).

Ten of 32 clinical variables were independently associated with RRT (Fig. 3b). They included hematological malignancy, IHD, onset of AKI in ICU, prior RRT for AKI, and higher delta-sCr. Others include surgical (versus medical) specialties, higher serum potassium and sCr at AKI detection, and higher baseline eGFR (all *p* < 0.05).

### AUROC of prediction models for RRT and mortality

**Fig. 4 a:**
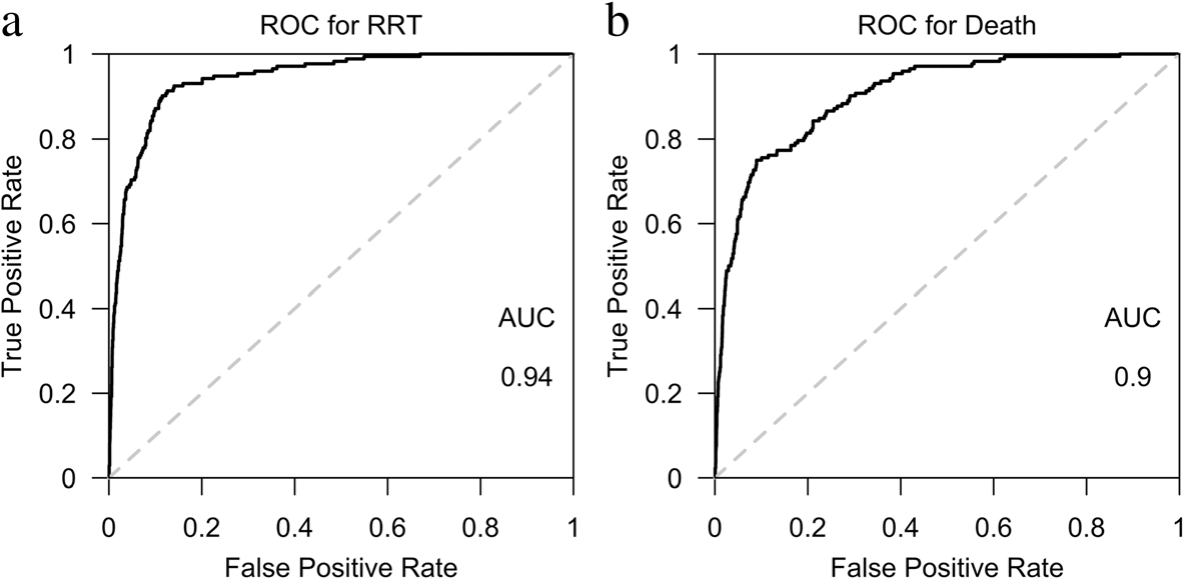
(AUROC for RRT), **Fig**. **4b** (AUROC for Mortality). Derived Area Under Receiver Operating Characteristic (AUROC) Curve of prediction models for progression to RRT (Fig. 4a) and mortality (Fig. 4b), using 32 clinical variables. For RRT prediction, the derived AUROC of the logistic regression model is 0.94, and the average AUROC after 10-fold out of sample cross validation is 0.93. The derived AUROC of the logistic regression model for mortality is 0.9, and after validation is also 0.9

The logistic regression models included all 32 intended clinical variables. For RRT prediction, the derived AUROC of the logistic regression model was 0.94, and the average AUROC after ten-fold out of sample cross validation was 0.93 (Fig. 4a).

The same set of variables was applied for mortality prediction. The derived AUROC of the logistic regression model for mortality was 0.9. The average AUROC after ten-fold out of sample cross validation was 0.9 (Fig. 4b).

**Fig. 5:**
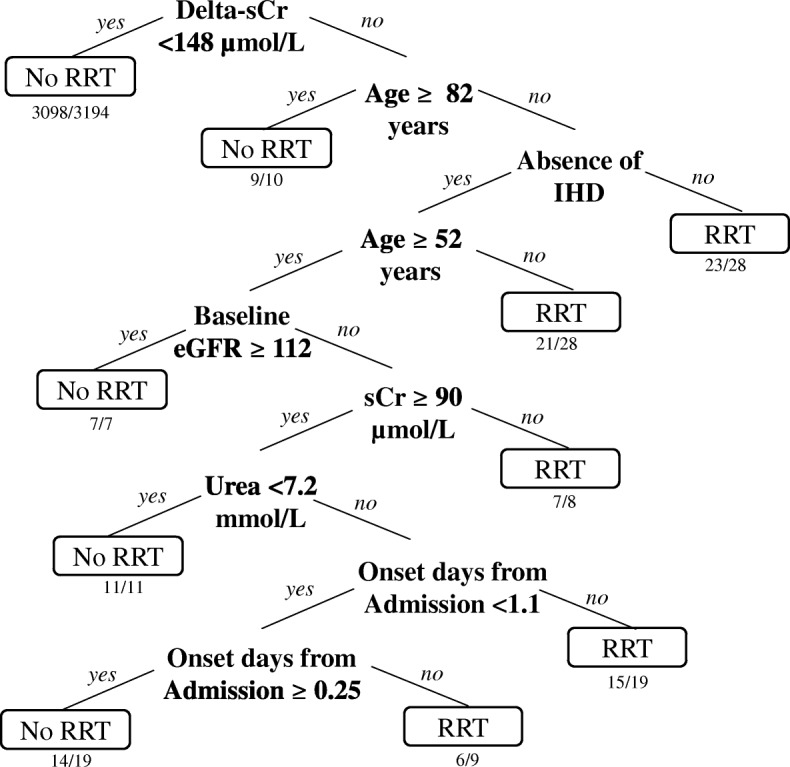
(Decision Tree Model for RRT). This can be used as a triage tool with probability of RRT calculated at each branch based on key risk factors. For example, individuals with delta-sCr > 148 μmol/L, younger than 82 years, and had IHD, 23 out of 28 of them required RRT. The factors identified that favoured decision for RRT included delta-sCr ≥ 148 μmol/L, younger age cut-offs, presence of IHD, baseline eGFR < 112 ml/min/1.73 m2, sCr and serum urea at AKI onset < 90 μmol/L and ≥ 7.2 mmol/L respectively, and different timings of AKI onset days from admission. Balanced accuracy of the model is 70.4%. Positive Predictive Value of 0.97, Negative Predictive Value of 0.78. RRT = Renal Replacement Therapy, IHD = Ischemic Heart Disease

The decision tree model for RRT achieved a balanced accuracy of 70.4%, with a positive predictive value of 0.97 and negative predictive value of 0.78 (Fig. 5). The factors identified that favored decision for RRT included delta-sCr ≥ 148 μmol/L, younger age cut-offs, presence of IHD, baseline eGFR ≤112 ml/min/1.73 m^2^, sCr and serum urea at AKI onset < 90 μmol/L and ≥ 7.2 mmol/L respectively, and different timings of AKI onset from admission. The decision tree model was not able to construct a binary tree with the 32 intended clinical variables for mortality.

## Discussion

In this prospective study involving 3333 hospitalized patients with AKI, we found a hospital mortality rate of 12% and RRT incidence of 5% following AKI. There were a significant number of EHR clinical variables that were associated with mortality in patients with all stages of AKI (KDIGO stages 1–3); Mortality predictors that were more specific for patients with more severe AKI, included admission under a medical specialty, higher baseline eGFR, higher serum potassium level on AKI diagnosis, higher delta-sCr, and acute diseases including intraabdominal infections, acute cardiac diseases, and hepatic decompensation. In addition, a number of EHR predictors including biochemical measures and sCr trajectory following AKI predicted RRT. The regression model for RRT prediction performed with an AUROC of more than 0.9. The key factors influencing RRT decision were delta sCr, age, IHD, baseline kidney function, and serum urea and creatinine levels at AKI detection. The model for mortality prediction also performed well with an AUROC of 0.9.

The ease of EHR-AKI detection allowed us to rapidly consolidate a hospital-wide database and build analytic models with more than 100,000 data points; as compared with prior AKI outcome prediction in the context of critical care involving simplified variable-list for ease of application [15]. The majority of our EHR data were indexed at AKI detection, with 74% of patients diagnosed at stage 1 AKI. This permits a lead-time for early intervention, as compared with other large prospective studies that predicted adverse outcomes in patients already receiving RRT at baseline [16, 17]. Many prior models were developed to predict AKI outcomes after cardiac surgery, [18, 19] but these may not be applicable to a wider hospital cohort. On the other hand, our prediction models were developed in hospital-wide patients. The models using EHR could be revised regularly with variable expansion to maintain currency, as validity of older clinical models are time-limited by the ever-changing healthcare profile [20]. The EHR prediction model is readily reproducible for customization in other healthcare systems, which is an important advantage over manual databases, as clinical models are unique for different institutions or regions with corresponding unique case-mix, which restricts the external validity of existing risk-stratification scores [21].

Our excellent RRT prediction model was contributed by deliberate inclusion of electronic clinical variables of prognostic interest. The key variables highlighted in the decision-tree analysis, especially delta sCr, suggest that an element of AKI trajectory is necessary in RRT prediction. This reflects the thought process of clinicians as we are guided by biochemical trends in addition to the patient’s clinical status when deciding on RRT. Other AKI trajectory elements explored in similar clinical models include blood urea nitrogen and sCr slope over 24 h, and sCr change from day before, with reported RRT prediction AUROCs of more than 0.8 [22, 23]. Current protein AKI biomarkers; ranging from urinary or plasma neutrophil gelatinase-associated lipocalin, urinary IL-18, to tissue inhibitor of metalloproteinases-2* insulin-like growth factor-binding protein 7, were reported in critical care or cardiac surgery settings to predict RRT or death, with AUROC of 0.6–0.85. [24–27]. Our superior clinical model for RRT prediction suggests that the composite of relevant clinical variables is equally important for model inclusion, in addition to biochemistry [22, 23].

The model performance for mortality also performed well. Key mortality predictors included common acute illnesses such as infections and cardiac diseases, as the precipitating causes of AKI are often the main determinant of mortality [28, 29]. The high mortality rate in cancer patients with AKI is well recognized and deserves intensified research for better diagnostics and preventive strategies to improve understanding of their mechanisms and outcomes [30, 31].

### Clinical significance and limitations

An early bundle care to aid clinical management upon electronic AKI detection was associated with reduced patient mortality and progression of AKI [32]. This bundle included interventions such as volume management, early control of sepsis, and cessation of nephrotoxins. An alert system that provides no risk stratification however, and flags up thousands of AKI patients per year, might be impractical, counter-productive and unable to influence ground practice or improve outcomes [33]. Our strong predictive models for RRT and mortality could potentially be used to develop or enhance an otherwise generic EHR-AKI alert, for clinicians to identify the highest-risk AKI patients, and this could allow more targeted and individualized interventions or prompt early nephrology consult. The greater adverse event rate of high-risk patients, also imply more feasible effect size for intervention trials, such as early versus conventional timing for RRT initiation. However, it is important to remember that prediction models simply provide the probability of outcomes and should be used as an adjunct to clinical decision making, and not to replace the latter. For instance, in our decision tree analysis for RRT (Fig. 5), patients aged > 82 years had lower probability of receiving RRT. This reflects the prevailing practice pattern but does not infer the lack of indication or benefit of RRT. Finally, we previously reported an aminoglycoside-associated AKI rate of 17% in the elderly, [34] and this was much higher than the current 4% all-cause AKI incidence in our institution. This emphasizes the need for intensified efforts to prevent drug-induced AKI.

Our study was single-centre in nature and consisted almost entirely of patients of Asian ethnicity. We only included unique patient AKI episodes to avoid over-representation by repeated covariates and events in a same patient but would have under-reported actual AKI event-rate. We did not have a non-AKI population for comparison, however our study was aimed at identifying mortality predictors in the AKI cohort. To overcome this limitation, we have done sub-cohort analysis for mortality predictors in mild versus severe AKI. We did not include urinary output criteria as that was not feasible using EHR outside of the critical care setting, but oliguria would have otherwise been an important covariate to aid RRT prediction. Our AKI trajectory element utilized in the prediction models was delta-sCr, and that is arguably not a definite early risk marker. Substitution of delta-sCr using an appropriate urinary biomarker tested at AKI onset could bridge this shortfall in effective outcome prediction and improve risk classification. [35, 36]. Another limitation of our study is the lack of data on need for outpatient chronic RRT following AKI during the hospitalization, which is an important patient outcome. Lastly, our RRT decision tree model was based on an in-vivo population and there was an imbalance between the binary outcomes with only 5% incidence of RRT. In order to overcome this limitation, we reported the “balanced accuracy” instead of the “overall accuracy” of the decision tree model, as an overall accuracy would lead to an optimistic estimate when tested on an imbalanced dataset, and reporting the “balanced accuracy” will reduce this bias [37].

## Conclusion

Our study presents an overview of in-hospital AKI epidemiology and highlights important clinical variables associated with RRT and mortality following AKI, which are routinely available in current electronic medical records. By utilizing EHR, AKI epidemiology and adverse outcomes can now be tracked regularly, with the potential for its use as a quality measure of inpatient management and care process. The EHR clinical models that we have developed for prediction of adverse outcomes are highly accurate, especially that for RRT prediction. These clinical tools are easily reproducible for real-time deployment, and may allow clinicians the ability to predict and focus on the highest-risk AKI sub-cohort for earlier, targeted intervention.

## Additional files

Additional file 1: **Table S1.** Table on acute disease categorisation. Patients’ acute disease diagnosis codes were based on ICD-9-CM (International Classification of Diseases, Ninth Revision, Clinical Modification) up till 2015, and SNOMED-CT (Systematized Nomenclature of Medicine - Clinical Terms) after 2015. We classified these diagnoses into 8 categories as shown in the table. These acute diseases were not mutually exclusive, and each patient may have one or more acute illnesses during the index hospitalization. (XLSX 11 kb)

Additional file 2: **Figure S1.** Figure on secondary outcomes. AKI stages based on modified KDIGO criteria at time of EHR-AKI detection, and peak AKI severity in stages. Proportion of patients admitted to Intensive Care Unit (ICU) on detection of AKI, and those who subsequently received ICU care, are shown. All figures are in percentages. (PPTX 45 kb)

Additional file 3: Multivariate analysis of mortality prediction in mild AKI and more severe AKI sub cohorts. Mortality predictors compared between patients with mild (peak KDGIO stage 1) AKI versus more severe (peak KDIGO stage 2 or 3) AKI described. (XLSX 12 kb)

## Abbreviations

AKI: Acute kidney injury
AUROC: Area under Receiver Operating Characteristic
CKD: Chronic kidney disease
CKD-EPI: CKD Epidemiology Collaboration
delta sCr: Serum Creatinine rise from AKI detection till peak Creatinine
EHR: Electronic health records
ESKD: End-stage kidney disease
ICU: Intensive care unit
IHD: Ischemic heart disease
RRT: Renal replacement therapy
sCr: Serum creatinine
