# Supplementary material for: Electronic health records accurately predict renal replacement therapy in acute kidney injury
Source: BMC Nephrol. 2019 Jan 31;20:32. doi: 10.1186/s12882-019-1206-4 (PMC6357378; doi:10.1186/s12882-019-1206-4)
Supplement: Supplementary file 2 — Figure S1. Figure on secondary outcomes. AKI stages based on modified KDIGO criteria at time of EHR-AKI detection, and peak AKI severity in stages. Proportion of patients admitted to Intensive Care Unit (ICU) on detection of AKI, and those who subsequently received ICU care, are shown. All figures are in percentages. (PPTX 45 kb) [file 12882_2019_1206_MOESM2_ESM.pptx]

## Slide 1
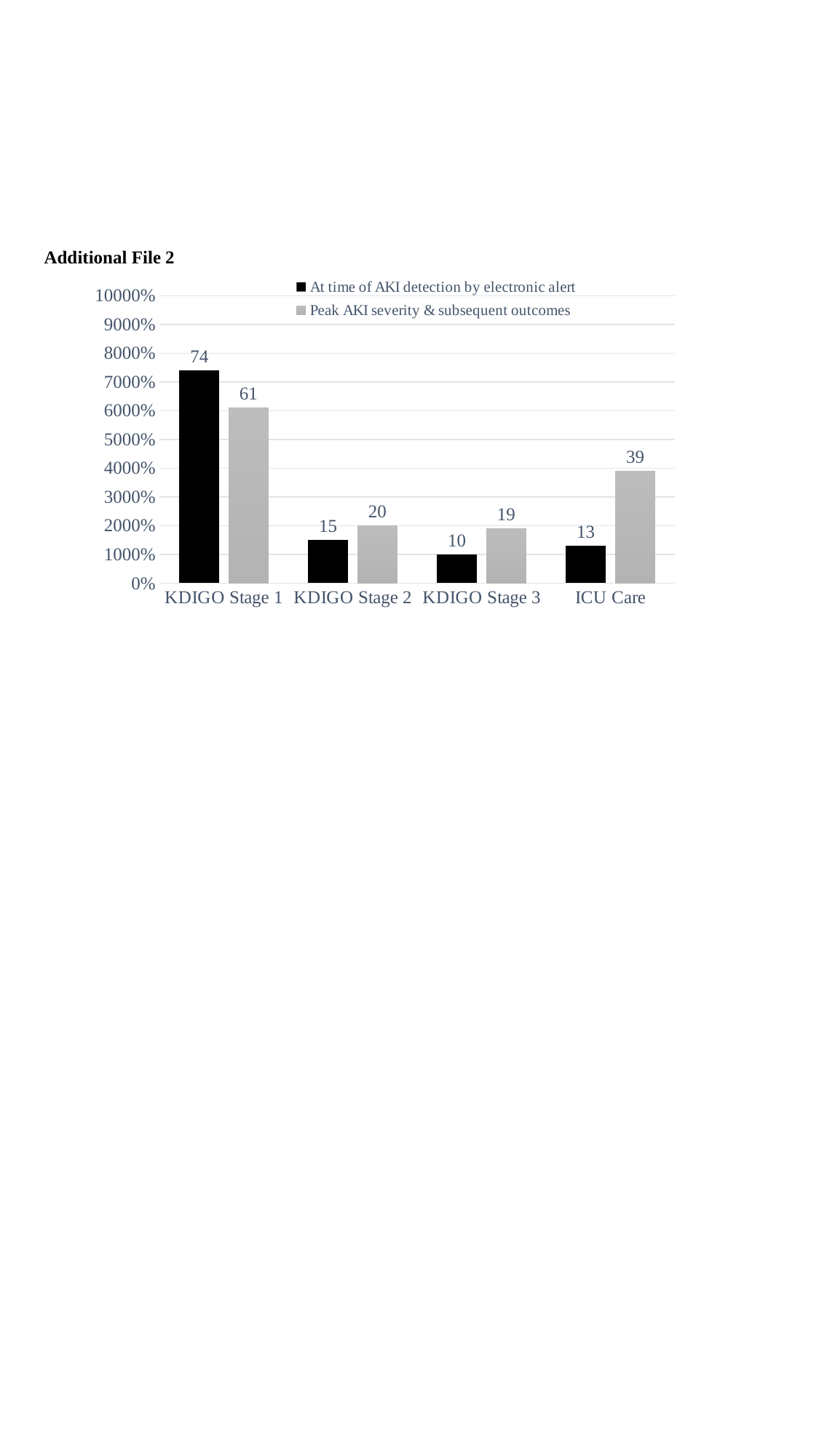

Additional File 2
### Chart
| Category | At time of AKI detection by electronic alert | Peak AKI severity & subsequent outcomes |
|---|---|---|
| KDIGO Stage 1 | 74.0 | 61.0 |
| KDIGO Stage 2 | 15.0 | 20.0 |
| KDIGO Stage 3 | 10.0 | 19.0 |
| ICU Care | 13.0 | 39.0 |
